# Supplementary material for: Extensive breaking of genetic code degeneracy with non-canonical amino acids
Source: Nat Commun. 2023 Aug 17;14:5008. doi: 10.1038/s41467-023-40529-x (PMC10435567; doi:10.1038/s41467-023-40529-x)
Supplement: Supplementary file 1 — Supplementary Information [file 41467_2023_40529_MOESM1_ESM.pdf]

Supplementary Information

Extensive breaking of genetic code degeneracy with non-canonical amino acids

Clinton A. McFeely[a,b], Bipasana Shakya[a,b], Chelsea A. Makovsky[a,b], Aiden K. Haney[a], T. Ashton Cropp[a], Matthew C. T. Hartman*[a,b]

[a]Department of Chemistry, Virginia Commonwealth University, 1001 W Main St., Richmond, VA 23284
[b]Massey Cancer Center, Virginia Commonwealth University, 401 College St. Richmond, VA 23219

*mchartman@vcu.edu

Table of Contents

Supplementary Table 1: sequences of oligonucleotides used to prepare mRNAs. 3

Supplementary Table 2: sequences of oligonucleotides used to prepare tRNAs. 4

Supplementary Table 3: observed vs expected masses for the leucine codon competitions using wt ribosomes. 6

Supplementary Table 4: observed vs expected masses for the leucine codon competitions using mS12 ribosomes 7

Supplementary Table 5: observed vs expected masses for the valine codon competition using t7tRNA and mS12 ribosomes. 8

Supplementary Figure 1: MALDI mass spectra of leucine codon competition translation reactions using wt ribosomes. 9

Supplementary Figure 2: analysis of the stoichiometry of the pre-charged tRNA mixtures used for the mS12 codon competitions. 10

Supplementary Figure 3: MALDI mass spectra of leucine codon competition translation reactions using mS12 ribosomes. 11

Supplementary Figure 4: Leucine codon competition using a mixture of wt and t7tRNA. 12

Supplementary Figure 5: MALDI-MS analysis of aaRS charging of a novel benzyl histidine ncAA with a mutant leucine aminoacyl tRNA synthetase. 13

Supplementary Figure 6: Sense codon reassignment with benzyl histidine at 15 µM results in mixed peptide products. 14

Supplementary Figure 7. In vitro translations with mRNAs containing 5 leucine codons but swapped tRNAs for 3-Fl-Leu and BzHis. 15

Supplementary Figure 8: urea-PAGE analysis of the in vitro transcribed valine tRNAs used in this paper. 16

Supplementary Figure 9: MALDI mass spectra of valine codon competition translation reactions using mS12 ribosomes. 17

Supplementary Figure 10: Valine sense codon reassignment using a third t7 Val tRNA shows failed orthogonality for the GUC codon. 18

Supplementary Figure 11: Absolute yield values for the translations described in Figure 5c. . 19

Supplementary Figure 12: MALDI-MS analysis of combined leucine and valine sense codon reassignment. 20

Supplementary References 21

mrNA 5 Leu codons p1	TTTACCGAAGGAGGAAAGAATGAAACTTTTGCTGT
mRNA 5 Leu codons p2	CGTCATCATCCTTATAATCTAGTAACAGCAAAAGTTTCATTCT
FWD primer	GGCGTAATACGACTCACTATAGGGTTAACTTTACCGAAGGAGGAAAGA
REV primer	CGAAGCTTATTTATCGTCATCATCCTTATAATCTAG
mRNA single Leu (CUC)	CCGAAGGAGGAAAGAATGAAACTCATGGATTATAAGGATGATGACGATAAATAATAG
mRNA single Leu (CUA)	CCGAAGGAGGAAAGAATGAAACTAATGGATTATAAGGATGATGACGATAAATAATAG
mRNA single Leu (UUA)	CCGAAGGAGGAAAGAATGAAATTAATGGATTATAAGGATGATGACGATAAATAATAG
mRNA singe Leu (CUU)	CCGAAGGAGGAAAGAATGAAACTTATGGATTATAAGGATGATGACGATAAATAATAG
mRNA single Leu (CUG)	CCGAAGGAGGAAAGAATGAAACTGATGGATTATAAGGATGATGACGATAAATAATAG
mRNA single Leu (UUG)	CCGAAGGAGGAAAGAATGAAATTGATGGATTATAAGGATGATGACGATAAATAATAG
FWD primer	GGCGTAATACGACTCACTATAGGGTTAACTTTACCGAAGGAGGAAAGA
REV primer	CTACTATTTGTCATCGTCG
mRNA single Val (GUU)	CCGAAGGAGGAAAGAATGAAAGTTAAAGATTATAAAGACGACGATG
mRNA single Val (GUC)	CCGAAGGAGGAAAGAATGAAAGTCAAAGATTATAAAGACGACGATG
mRNA single Val (GUA)	CCGAAGGAGGAAAGAATGAAAGTAAAAGATTATAAAGACGACGATG
mRNA single Val (GUG)	CCGAAGGAGGAAAGAATGAAAGTGAAAGATTATAAAGACGACGATG
FWD primer	GGCGTAATACGACTCACTATAGGGTTAACTTTACCGAAGGAGGAAAGA
REV primer	CTACTATTTGTCATCGTCGTCTTTATAATC
mrNA Leu/Val combo 1 p1	TTTACCGAAGGAGGAAAGAATGAAACTTGTGCTA
mRNA Leu/Val combo 1 p2	ATCGTCATCATCCTTATAATCTAGCACAAGTTTCATTCTTTC
mRNA Leu/Val combo 2 p1	TTTACCGAAGGAGGAAAGAATGAAACTCGTACTG
mRNA Leu/Val combo 2 p2	ATCGTCATCATCCTTATAATCCAGTACGAGTTTCATTCTTTC
mRNA Leu/Val combo 3 p1	TTTACCGAAGGAGGAAAGAATGAAATTAGTCTTG
mRNA Leu/Val combo 3 p2	ATCGTCATCATCCTTATAATCCAAGACTAATTTCATTCTTTC
FWD primer	GGCGTAATACGACTCACTATAGGGTTAACTTTACCGAAGGAGGAAAGA
REV primer	CGAAGCTTATTTATCGTCATCATCCTTATAAT
mRNA Triple Val template	CCGAAGGAGGAAAGAATGAAAGTAGCCGCAGTCGCCGCAGTGAAAGATTATAAAGACGAC
FWD primer	GGCGTAATACGACTCACTATAGGGTTAACTTTACCGAAGGAGGAAAGA
REV primer	CTACTATTTGTCATCGTCG

Supplementary Table 1: sequences of oligonucleotides used to prepare mRNAs. PCR primers are listed from the 5’ to 3’ end. PCR was conducted in one pot between a template, a forward primer, and a reverse primer in all cases except for the 5 leucine codon mRNA and the Leu/Val combination mRNAs, for which two primers (p1 and p2) were used in PCR to make a template which was subsequently used with forward and reverse primers to make the full-length PCR products. Select designs include a single 2’-O methylated guanine base to reduce non-templated nucleotide addition at the 3’ end of the PCR products.

tRNA Leu 1 p1	GTAATACGACTCACTATAGCGAAGGTGGCGGAATTGGTAGACGCGCTAGCTTCA
tRNA Leu 1 p2	CCCACGTCCGTAAGGACACTAACACCTGAAGCTAGCGCGTC
tRNA Leu 1 p3	TGGTGCGAGGGGGGGGACTTGAACCCCCACGTCCGTAAG
tRNA Leu 1 p4	GTAATACGACTCACTATAGC
tRNA Leu 1 p5	TGGTGCGAGGGG
tRNA Leu 2 p1	GTAATACGACTCACTATAGCCGAGGTGGTGGAATTGGTAGACACGCTACC
tRNA Leu 2 p2	CCGTAAGCCCTATTGGGCACTACCACCTCAAGGTAGCGTGTCTACCA
tRNA Leu 2 p3	TGGTACCGAGGACGGGACTTGAACCCGTAAGCCCTATTGG
tRNA Leu 2 p4	GTAATACGACTCACTATAGCC
tRNA Leu 2 p5	TGGTACCGAGGACGG
tRNA Leu 3 p1	GTAATACGACTCACTATAGCGGGAGTGGCGAAATTGGTAGACGCACCAGATTTA
tRNA Leu 3 p2	GAACTCGCACACCTTGCGGCGCCAGAACCTAAATCTGGTGCGTCTACCA
tRNA Leu 3 p3	TGGTGCGGGAGGCGAGACTTGAACTCGCACACCTTG
tRNA Leu 3 p4	GTAATACGACTCACTATAGCGG
tRNA Leu 3 p5	TGGTGCGGGAGGCGA
tRNA Leu 4 p1	GTAATACGACTCACTATAGCCGAAGTGGCGAAATCGGTAGACGCAGTTGATTCA
tRNA Leu 4 p2	GAACCGGCACGTATTTCTACGGTTGATTTTGAATCAACTGCGTCTACCG
tRNA Leu 4 p3	TGGTGCCGAAGGCCGGACTCGAACCGGCACGTA
tRNA Leu 4 p4	GTAATACGACTCACTATAGC
tRNA Leu 4 p5	TGGTGCCGAAGGCC
tRNA Leu 5 p1	GTAATACGACTCACTATAGCCCGGATGGTGGAATCGGTAGACACAAGGGATTTA
tRNA Leu 5 p2	CGCACAGCGCGAACGCCGAGGGATTTTAAATCCCTTGTGTCTACCG
tRNA Leu 5 p3	TGGTACCCGGAGCGGGACTTGAACCCGCACAGCGC
tRNA Leu 5 p4	GTAATACGACTCACTATAGCCCG
tRNA Leu 5 p5	TGGTACCCGGAGC
tRNA Val 1 mut. p1	GTAATACGACTCACTATAGGGTGATTAGCTCAGCTGGGAGAGCACCTCC
tRNA Val 1 mut. p2	CGCCGACCCCCTCCTTGTGAGGGAGGTGCTCTCCCA
tRNA Val 1 mut. p3	TGGTGGGTGATGACGGGATCGAACCGCCGACCCCCTC
tRNA Val 1 mut. p4	GTAATACGACTCACTATAGGGTG
tRNA Val 1 mut. p5	TGGTGGGTGATGACGGGA
tRNA Val 1 p1	GTAATACGACTCACTATAGGGTGATTAGCTCAGCTGGGAGAGCACCTCCC
tRNA Val 1 p2	GAACCGCCGACCCCCTCCATGTAAGGGAGGTGCTCTCCCAGCT
tRNA Val 1 p3	TGGTGGGTGATGACGGGATCGAACCGCCGACCCCCTCC
tRNA Val 1 p4	GGCGTAATACGACTCACTATAG
tRNA Val 1 p5	TGGTGGGTGATGACGGGATC
tRNA Val 2 p1	GGCGTAATACGACTCACTATAGCGTTCATAGCTCAGTTGGTTAGAGCAC
tRNA Val 2 p2	CCAACGACCCCCACCATGTCAAGGTGGTGCTCTAACCAACTGAG
tRNA Val 2 p3	TGGTGCGTTCAATTGGACTCGAACCAACGACCCCCACCA
tRNA Val 2 p4	GGCGTAATACGACTCACTATAG
tRNA Val 2 p5	TGGTGCGTTCAATTGGA

Supplementary Table 2: sequences of oligonucleotides used to prepare tRNAs.
PCR primers are listed from the 5' to 3' end. PCR was conducted in three steps: an extension between primers 1 and 2, PCR 1 between primers 3 and 4, and PCR 2 between primers 4 and 5. Each PCR product was designed to include a single 2'-O methylated guanine base to reduce non-templated nucleotide addition at the 3' terminus.

	$_{d3}$**Leu**^{CAG}	$_{d7}$**Leu**^{GAG}	$_{d0}$**Leu**$^{VAG/UAG}$	$_{d17}$**Leu**$^{BAA/CAA}$	$_{d10}$**Leu**$^{)AA/UAA}$
Expected	1,547.68	1,551.71	1,544.66	1,561.67	1,554.72
CUA rxn 1 observed (wt)	1,547.32	1,551.28	1,544.31	Not observed	1,554.37
CUA rxn 2 observed (wt)	1,547.40	1,551.36	1,544.39	Not observed	1,554.45
CUA rxn 3 observed (wt)	1,547.56	1,551.52	1,544.47	Not observed	1,554.61
UUA rxn 1 observed (wt)	Not observed	Not observed	1,544.63	1,561.69	1,554.53
UUA rxn 2 observed (wt)	Not observed	Not observed	1,544.55	1,561.68	1,554.61
UUA rxn 3 observed (wt)	Not observed	Not observed	1,544.39	1,561.53	1,554.54
CUA rxn 1 observed (t7)	1,547.70	1,551.74	1,544.77	Not observed	Not observed
CUA rxn 2 observed (t7)	1,547.56	1,551.68	1,544.63	Not observed	Not observed
CUA rxn 3 observed (t7)	1,547.72	1,551.76	1,544.71	Not observed	Not observed
UUA rxn 1 observed (t7)	Not observed	Not observed	Not observed	1,561.69	1,554.78
UUA rxn 2 observed (t7)	Not observed	Not observed	Not observed	1,561.85	1,554.70

UUA rxn 3 observed (t7)	Not observed	Not observed	Not observed	1,561.77	1,554.78

Supplementary Table 3: observed vs expected masses for the leucine codon competitions using wt ribosomes

Analysis of the mass spectra from the CUA and UUA codon competitions (each performed in triplicate) reveals the observed masses of peaks associated with codon readthrough by one of five isotopically labelled tRNAs. In cases where readthrough of a codon by a tRNA was less than 5%, the observed mass was marked as not observed.

	${}_{d3}Leu^{CAG}$	${}_{d7}Leu^{GAG}$	${}_{d0}Leu^{VAG/UAG}$	${}_{d17}Leu^{BAA/CAA}$	${}_{d10}Leu^{)AA/UAA}$
Expected	1,547.68	1,551.71	1,544.66	1,561.67	1,554.72
CUA rxn 1 observed (wt)	1,547.87	Not observed	1,544.78	Not observed	Not observed
CUA rxn 2 observed (wt)	1,547.95	Not observed	1,544.94	Not observed	Not observed
CUA rxn 3 observed (wt)	1,547.87	Not observed	1,544.86	Not observed	Not observed
UUA rxn 1 observed (wt)	Not observed	Not observed	Not observed	1,561.79	1,554.93
UUA rxn 2 observed (wt)	Not observed	Not observed	Not observed	1,562.00	1,554.85
UUA rxn 3 observed (wt)	Not observed	Not observed	Not observed	1,561.53	1,554.77
CUA rxn 1 observed (t7)	Not observed	Not observed	1,544.69	1,561.99	Not observed

CUA rxn 2 observed (t7)	Not observed	Not observed	1,544.77	1,561.91	Not observed
CUA rxn 3 observed (t7)	Not observed	Not observed	1,544.77	1,561.67	Not observed
UUA rxn 1 observed (t7)	Not observed	Not observed	1,544.93	Not observed	1,554.92
UUA rxn 2 observed (t7)	Not observed	Not observed	1,544.85	Not observed	1,554.76
UUA rxn 3 observed (t7)	Not observed	Not observed	1,544.85	Not observed	1,554.84

Supplementary Table 4: observed vs expected masses for the leucine codon competitions using mS12 ribosomes

Analysis of the mass spectra from the CUA and UUA codon competitions (each performed in triplicate) reveals the observed masses of peaks associated with codon readthrough by one of five isotopically labelled tRNAs. In cases where readthrough of a codon by a tRNA was less than 5%, the observed mass was marked as not observed.

	$_{d0}$Val^{UAC}	$_{d8}$Val^{GAC}
Expected	1,527.69	1,535.75
GUU rxn 1 observed	1,527.38	1,535.41
GUU rxn 2 observed	1,527.22	1,535.33
GUU rxn 3 observed	1,527.14	1,535.33
GUA rxn 1 observed	1,527.38	1,535.49
GUA rxn 2 observed	1,527.30	1,535.41
GUA rxn 3 observed	1,527.22	1,535.33
GUC rxn 1 observed	1,527.22	1,535.33
GUC rxn 2 observed	1,528.00	1,535.25
GUC rxn 3 observed	1,528.08	1,535.09
GUG rxn 1 observed	1,526.91	1,535.09
GUG rxn 2 observed	1,526.83	1,535.01
GUG rxn 3 observed	1,526.59	Not observed

Supplementary Table 5: observed vs expected masses for the valine codon competition using t7tRNA and mS12 ribosomes.
Analysis of the mass spectra from the valine codon competitions (each performed in triplicate) reveals the observed masses of peaks associated with codon readthrough by one of two isotopically labelled tRNAs. In cases where readthrough of a codon by a tRNA was less than 5%, the observed mass was marked as not observed.

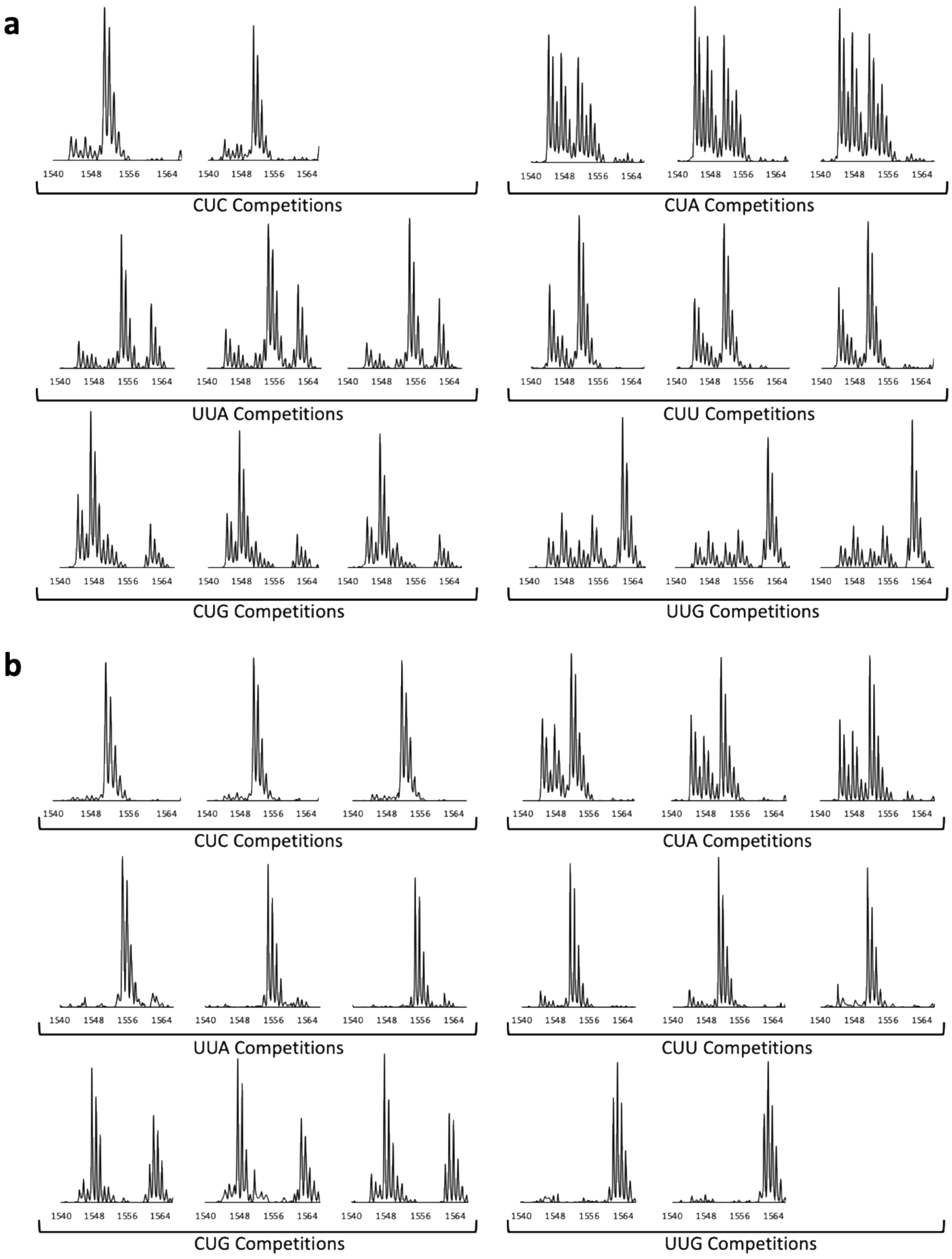

Supplementary Figure 1: MALDI mass spectra of leucine codon competition translation reactions using wt ribosomes.

Codon competition translation reactions were performed against all six leucine codons, using either five wild type leucyl tRNAs (a) or five synthetic leucyl tRNAs (b). Reactions were performed in triplicate, except for the CUC – wt tRNA and UUG – t7tRNA pairings, which were performed in duplicate. Translations were performed for 30 minutes at 37 °C, with pre-charged tRNAs added to a final concentration of 5 µM and wt ribosomes at 5 µM.

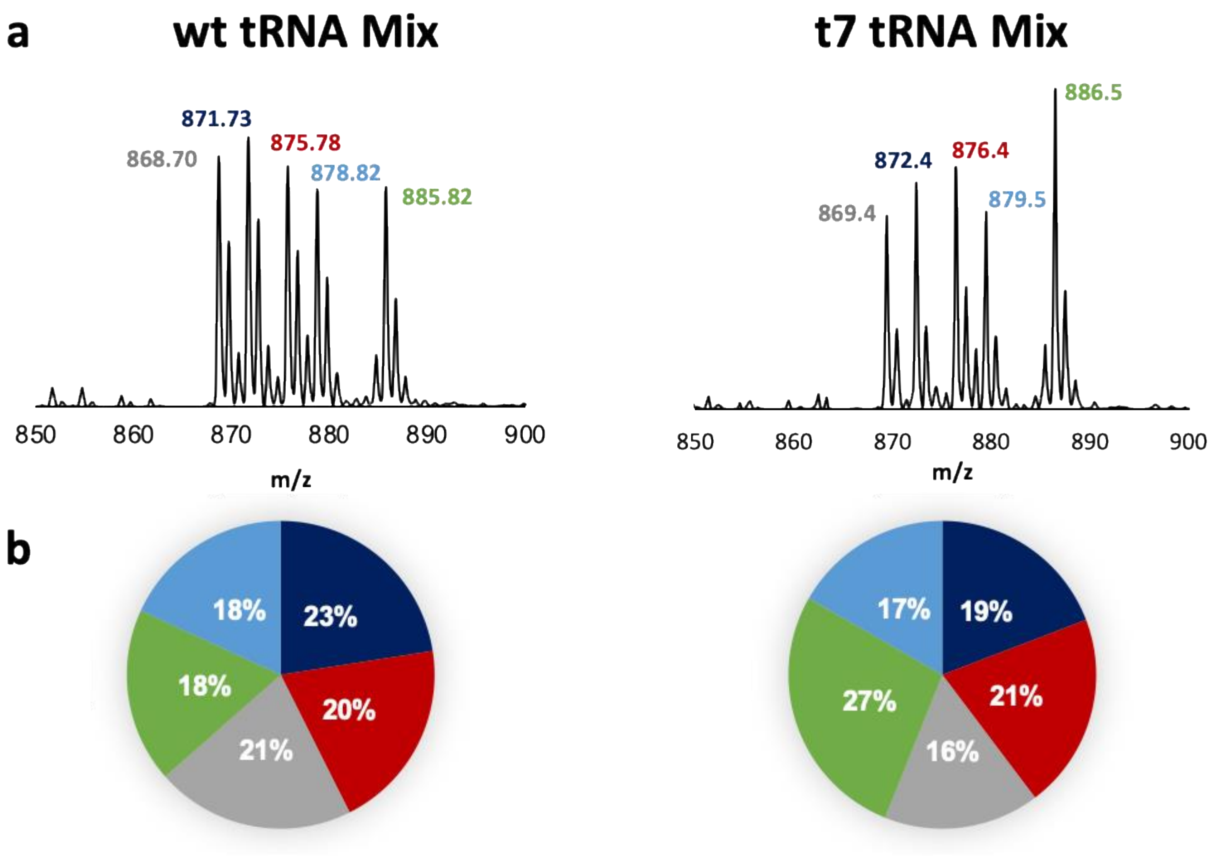

Supplementary Figure 2: analysis of the stoichiometry of the pre-charged tRNA mixtures used for the mS12 codon competitions.

(a) Mass spectra analysis of the five isotopically labelled leucyl tRNAs reveals their stoichiometric balance (b).

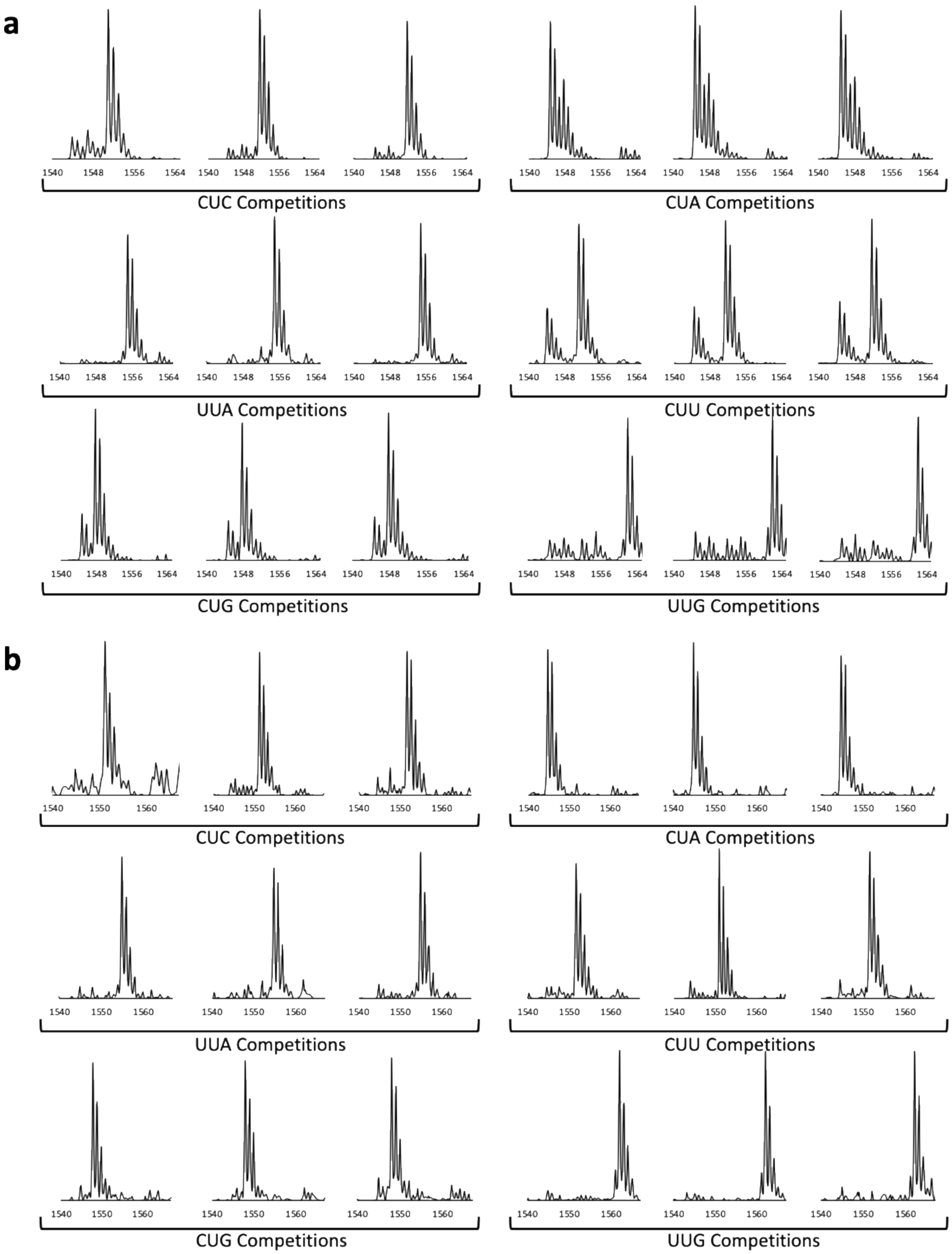

Supplementary Figure 3: MALDI mass spectra of leucine codon competition translation reactions using mS12 ribosomes.

Codon competition translation reactions were performed against all six leucine codons, using either five wild type leucyl tRNAs (a) or five synthetic leucyl tRNAs (b). Reactions were performed in triplicate. Translations were performed for 30 minutes at 37 °C, with pre-charged tRNAs added to a final concentration of 5 µM and mS12 ribosomes at 1.2 µM.

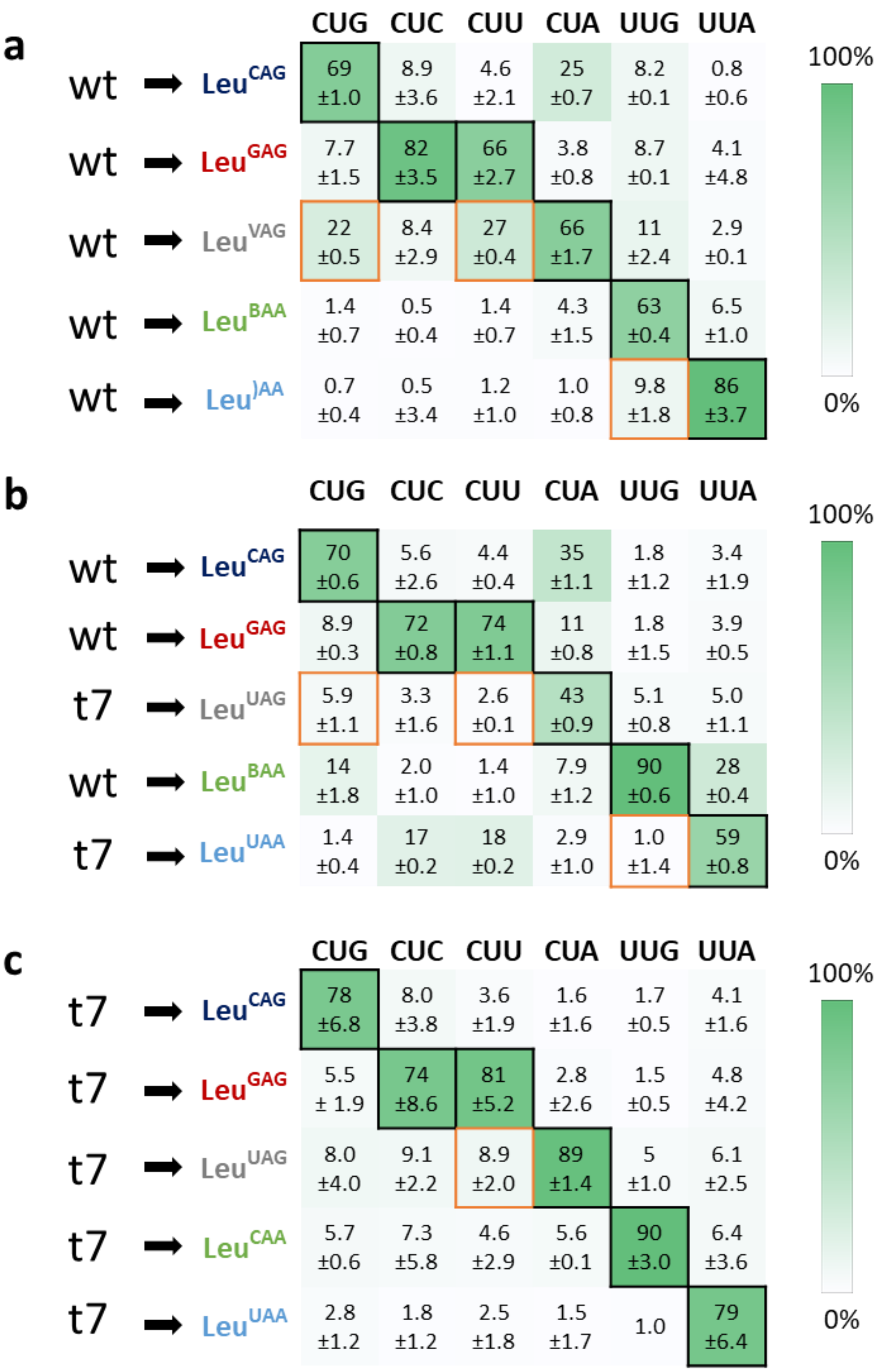

Supplementary Figure 4: Leucine codon competition using a mixture of wt and t7tRNA.

Codon competition experiments performed between five isotopically labelled leucyl tRNAs, either all wt (a), a wt/t7 mixture (b), or all t7tRNA (c) using mS12 hyper-accurate ribosomes. In these heat maps, darker green corresponds with higher readthrough. Black borders represent reported cognate codon readthrough (Watson Crick, with the exception of CUU) and orange

borders represent reported wobble pairing readthrough.[1] Translations were performed for 30 minutes at 37 °C, with all pre-charged AA-tRNAs at a final concentration of 5 µM.

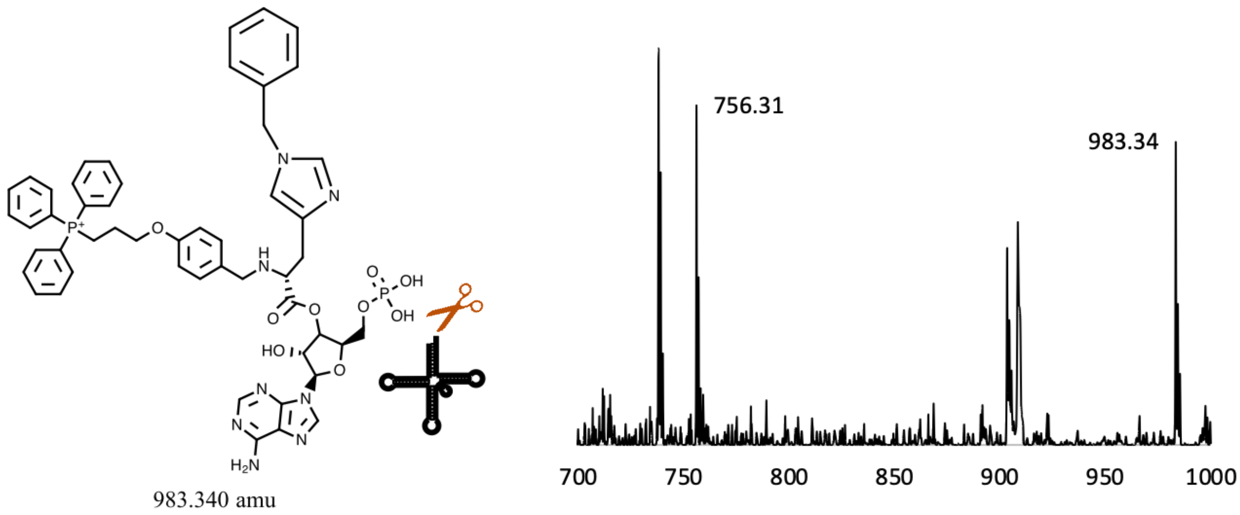

Supplementary Figure 5: MALDI-MS analysis of aaRS charging of a novel benzyl histidine ncAA with a mutant leucine aminoacyl tRNA synthetase.

Benzyl histidine was pre-charged onto tRNA Leu^{CAG} as described in the methods for two hours at 37 °C with a mutant leucine aminoacyl tRNA synthetase, prior to derivatization and digestion using nuclease P1 leaving the derivatized AA-AMP (left) for mass spectrometry analysis (right).[2] The final concentration of benzyl histidine in the assay was 5 mM. The expected mass is shown below the derivatized structure, with the observed mass shown in the mass spectrum. 4-formylphenoxypropyl triphenylphosphonium AMP is an expected byproduct, with an expected m/z of 756.23 Da and observed mass of 756.31 Da.[2]

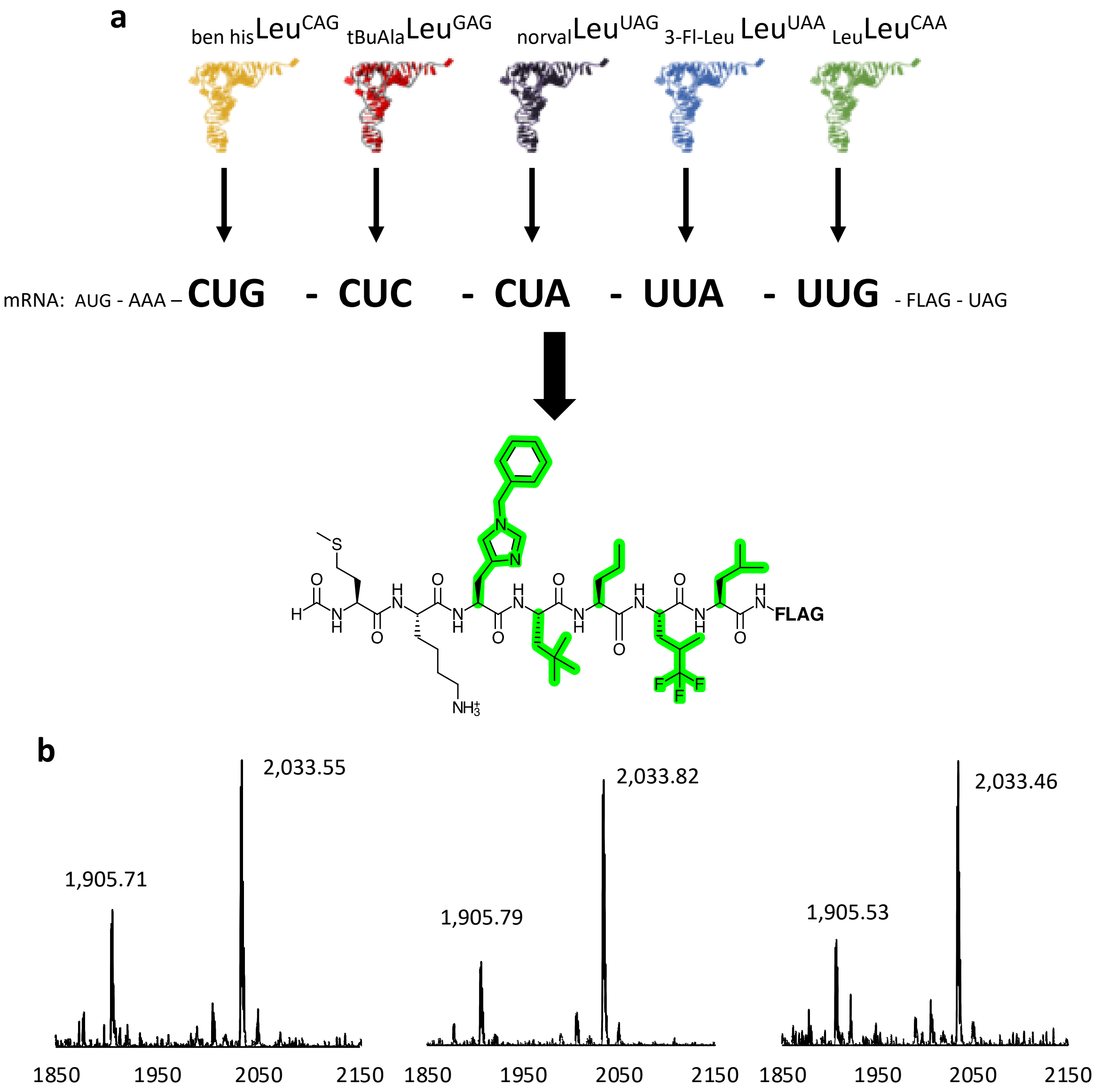

Supplementary Figure 6: Sense codon reassignment with benzyl histidine at 15 µM results in mixed peptide products.

(a) a single mRNA with five adjacent leucine codons is decoded in the PURE translation system by five pre-charged leucine tRNA isoacceptors, each bearing a distinct leucine analog (benzyl histidine, tert-butyl alanine, norvaline, tri-fluoro leucine, and leucine) to translate the peptide of interest. Amino acids encoded by the reassigned codons are highlighted in green. S12 ribosomes and t7tRNA were used. Translations were performed for 30 minutes at 37 °C, with all pre-charged AA-tRNAs at a final concentration of 15 µM. (b) Mass spectra of the translation results, performed in triplicate, shows major peaks matching the expected mass of 2,033.95 Da, with minor peaks (left) matching the mass of a norvaline misincorporation. This misincorporation occurs when norval-Leu^{UAG} steals the CUG codon from BzHis-Leu^{CAG}.

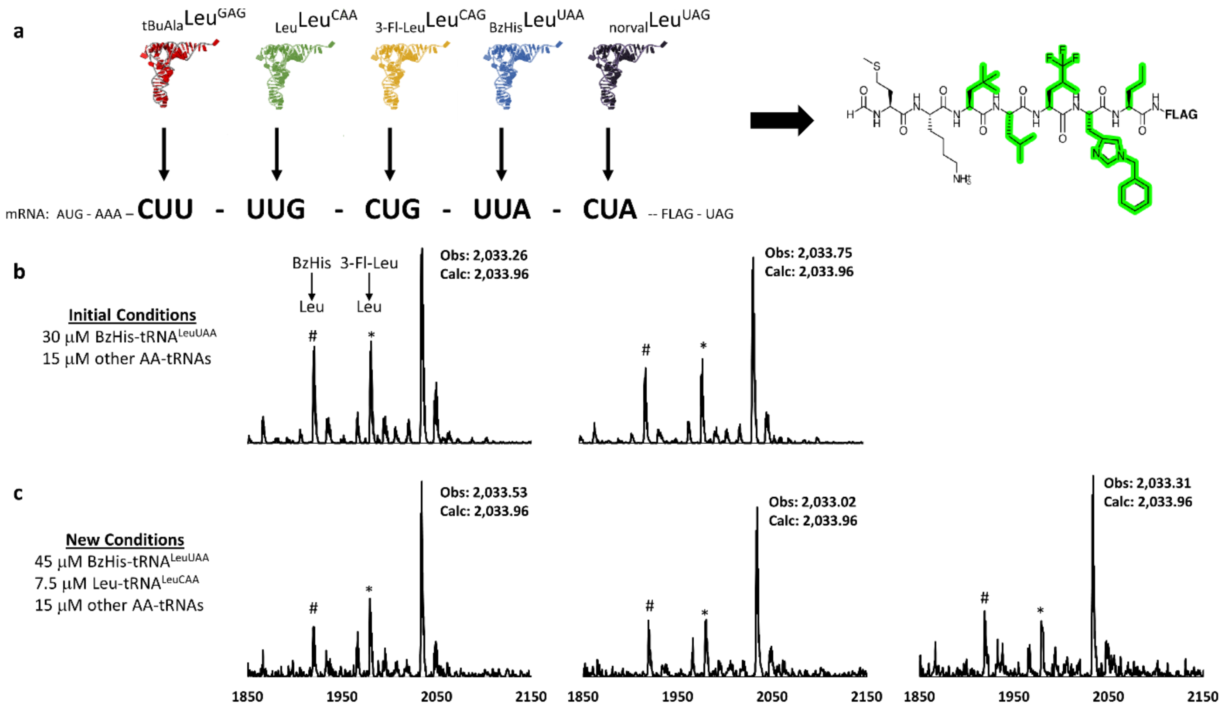

Supplementary Figure 7. In vitro translations with mRNAs containing 5 leucine codons but swapped tRNAs for 3-Fl-Leu and BzHis.

(a) Codon scheme showing the re-encoding strategy and the expected peptide product. (b) MALDI-MS of translations (30 min, 37 °C) with AA-tRNAs added at the concentrations shown. The major misincorporation peaks are labeled. # Leu for BzHis, Calc. 1919.93 Obs: 1919.29 (left) and 1919.77 (right); * Leu for 3-Fl-Leu, Calc. 1979.98, Obs: 1979.30 (left) and 1979.80 (right). (c) MALDI-MS of translations (30 min, 37 °C) with the added AA-tRNAs shown showing improved yield of the expected peptide. The major misincorporation peaks are labeled. # Leu for BzHis, Calc. 1919.93. Obs: 1919.57 (left), 1919.11 (middle), and 1919.27 (right); * Leu for 3-Fl-Leu, Calc. 1979.98, Obs: 1979.48 (left), 1979.07 (middle), 1979.36 (right).

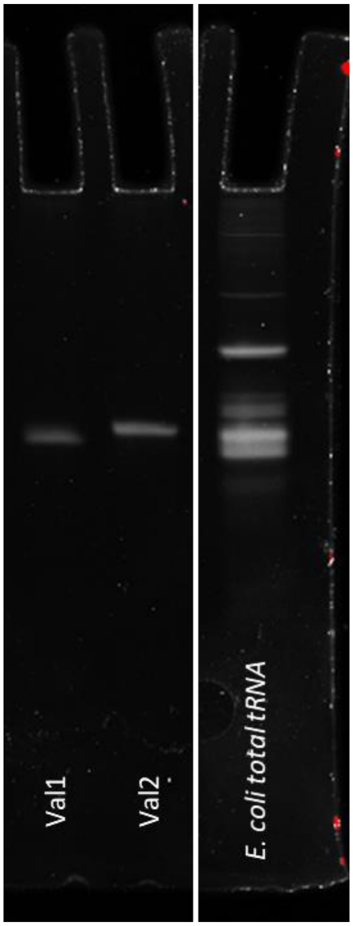

Supplementary Figure 8: urea-PAGE analysis of the in vitro transcribed valine tRNAs used in this paper.

Analytical urea-PAGE gel used to analyze the t7-transcribed valine tRNAs used in the paper with *E. coli* total tRNA used as a reference. The two images are non-adjacent lanes from the same gel. Val1 is expected to be 76 bp, and Val2 is expected to be 77 bp. The majority of *E. coli* tRNAs are 76 or 77 bp. This gel is representative of duplicate experiments.

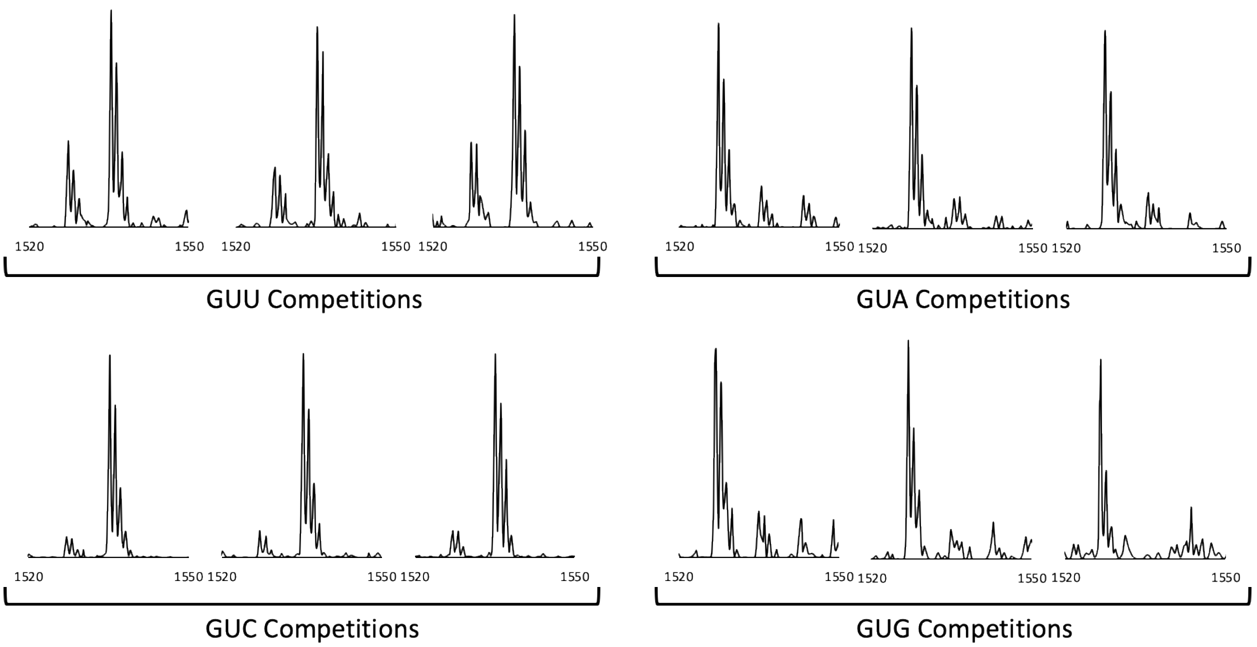

Supplementary Figure 9: MALDI mass spectra of valine codon competition translation reactions using mS12 ribosomes.

Codon competition translation reactions were performed against all four valine codons, using two synthetic valine tRNAs. Reactions were performed in triplicate. Translations were performed for 30 minutes at 37 °C, with all pre-charged AA-tRNAs at a final concentration of 10 μM.

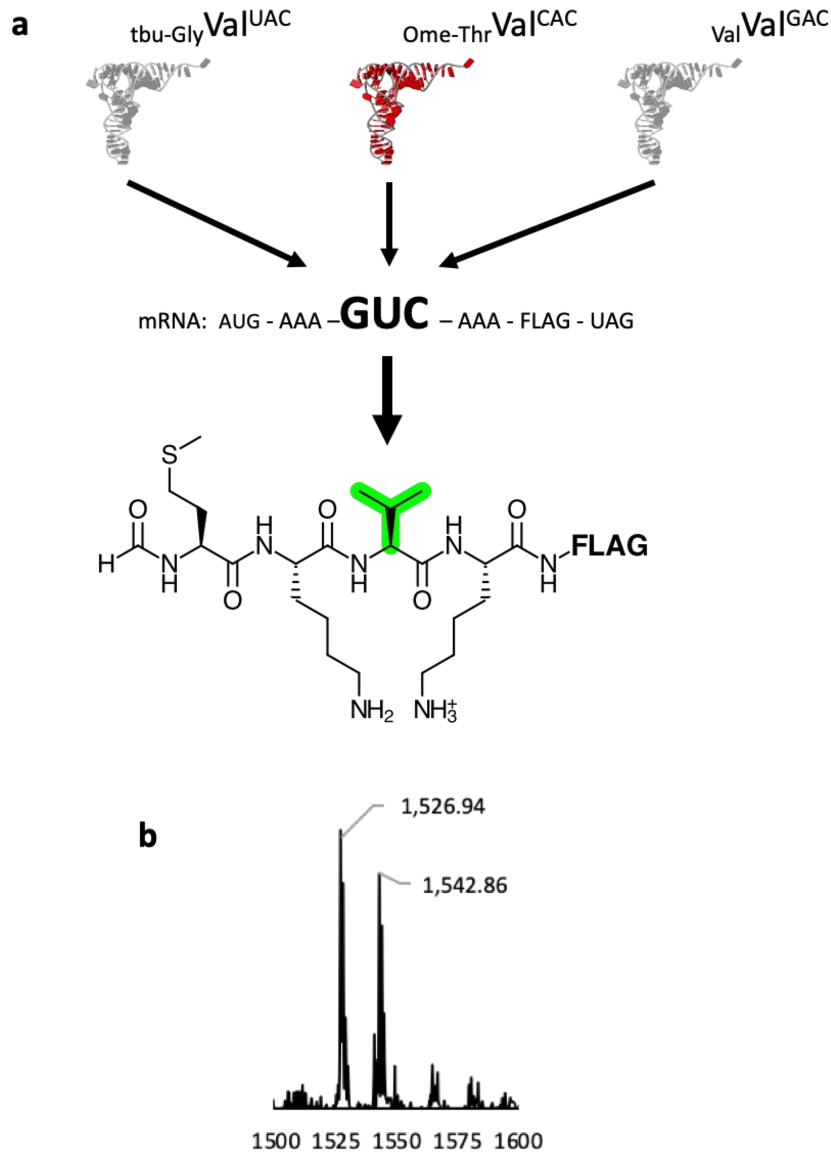

Supplementary Figure 10: Valine sense codon reassignment using a third t7 Val tRNA shows failed orthogonality for the GUC codon.

(a) Reassignment of the GUC valine codon using a pre-charged mixture of t7tRNA Val^{UAC}, Val^{CAC}, and Val^{GAC} charged with tert-butyl glycine, O-methyl threonine, and valine respectively. The desired readthrough was by Val^{GAC}, resulting in the translation of a peptide bearing valine (expected mass 1,527.70 Da). (b) MALDI-MS analysis revealed mixed peptide products: one with an observed mass of 1,526.94 Da indicating the translation of the desired peptide, and another with an observed mass of 1,542.86 Da, matching the mass of a peptide bearing OMe-Thr, indicating readthrough by the Val^{CAC} mutant. The translation (30 µL) was performed for 30 minutes at 37 °C with the pre-charged tRNAs added to a final concentration of 10 µM.

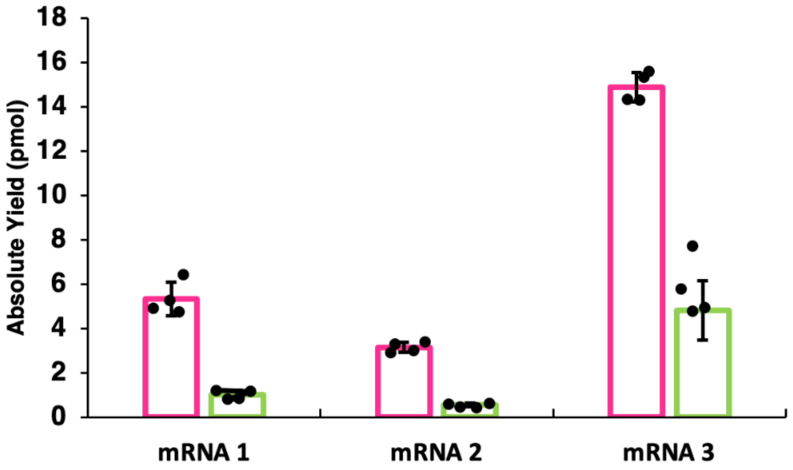

Supplementary Figure 11: Absolute yield values for the translations described in Figure 5c.

Yields were from 30 μL in vitro translation reactions carried out for 30 min under the conditions described in Fig. 5 and were measured by ^{35}S-Methionine capture onto anti-FLAG agarose beads.

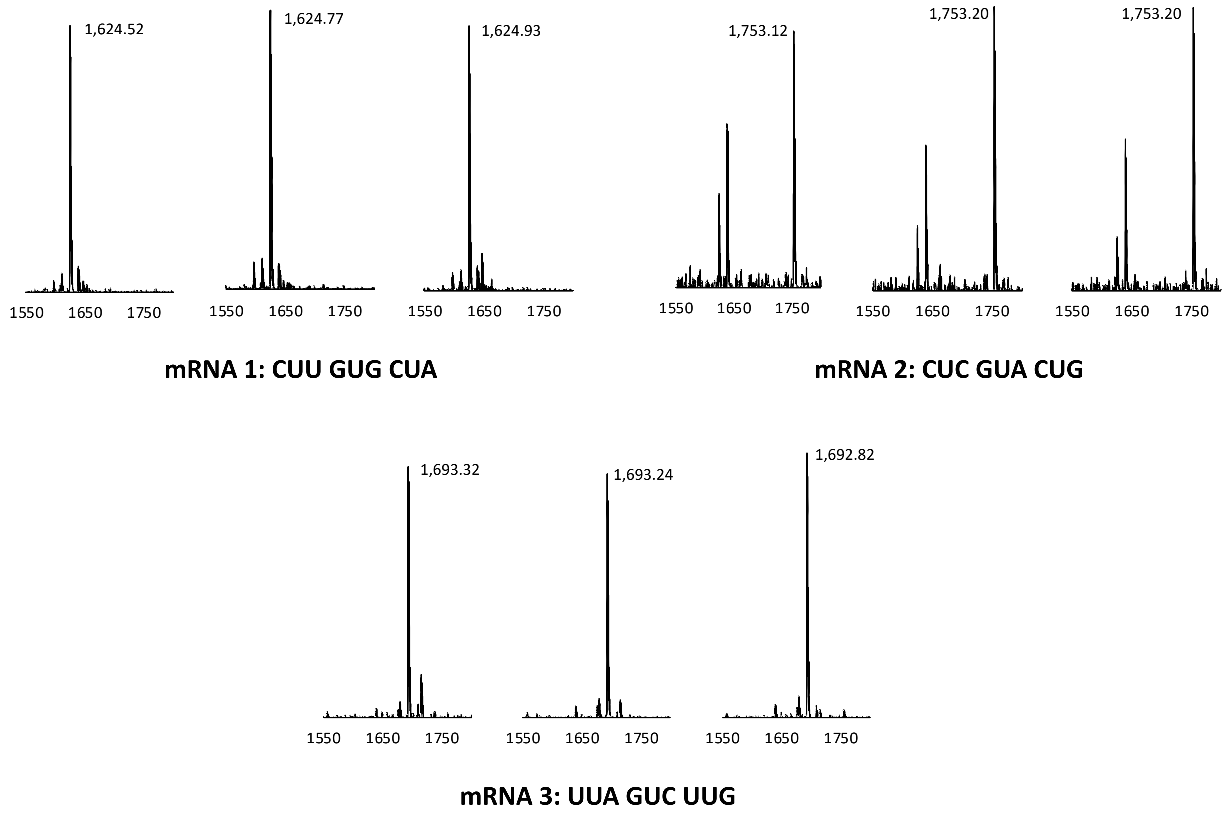

Supplementary Figure 12: MALDI-MS analysis of combined leucine and valine sense codon reassignment.

Mass spectra analyzing the translation results using each of the three mRNAs. The 30 µL translations reactions were incubated for 30 minutes at 37 °C with final concentrations of the pre-charged valine tRNAs at 10 µM, BzHis-tRNA Leu^{CAG} at 30 µM, and the remaining Leu tRNAs at 15 µM. Observed masses are shown next to each major peak (expected masses for mRNA 1, 2, 3 are 1,625.77 Da, 1,753.81 Da, and 1,693.76 Da respectively). In the mRNA 2 mass spectra, a BzHis to norval misincorporated peptide peak is the second tallest peak, and a peptide with BzHis to tBuGly misincorporations is the smallest observable peak.

Supplementary References

(1) Cui, Z.; Stein, V.; Tnimov, Z.; Mureev, S.; Alexandrov, K. Semisynthetic TRNA Complement Mediates in Vitro Protein Synthesis. *J. Am. Chem. Soc.* **2015**, *137* (13), 4404–4413. https://doi.org/10.1021/ja5131963.

(2) Hartman, M. C. T.; Josephson, K.; Szostak, J. W. Enzymatic Aminoacylation of TRNA with Unnatural Amino Acids. *Proc. Natl. Acad. Sci. U. S. A.* **2006**, *103* (12), 4356–4361. https://doi.org/10.1073/pnas.0509219103.
